# A Qualitative Exploration of Practitioners’ Understanding of and Response to Child-to-Parent Aggression

**DOI:** 10.1177/0886260520967142

**Published:** 2020-10-26

**Authors:** Sarah E. O’Toole, Stella Tsermentseli, Athanasia Papastergiou, Claire P. Monks

**Affiliations:** 1 University College London, UK; 2 University of Greenwich, London, UK; 3 Bangor University, Gwynedd, UK

**Keywords:** child-to-parent aggression, family violence, parent abuse, qualitative, practitioners

## Abstract

There has been limited research and policy directed toward defining and understanding child-to-parent aggression (CPA), resulting in inconsistent definitions, understandings, and responses, which has a detrimental impact on families. In particular, there have been limited qualitative studies of those working on the frontline of CPA, hindering the development of effective policy. The present qualitative study therefore aimed to explore practitioner perspectives of CPA. Twenty-five practitioners from diverse fields (e.g., youth justice, police, charities) participated in four focus groups relating to their experiences of working with CPA in the United Kingdom. Thematic analysis of focus groups revealed three key themes: definitions of CPA, understanding of CPA risk factors, and responding to CPA. Practitioners understood CPA to be a broad use of aggression to intimidate and control parents and highlighted a range of individual (e.g., mental health, substance abuse) and social (e.g., parenting, gangs) risk factors for CPA. Further, practitioners felt that current methods of reporting CPA were ineffective and may have a detrimental impact on families. The findings of this study have implications for CPA policy and support the need for a multiagency and coordinated strategy for responding to CPA.

Despite reports of child-to-parent aggression (CPA) increasing in prevalence (Coogan, 2012), CPA has received limited research attention in comparison to other forms of interpersonal violence (Condry & Miles, 2013). Referred to by a variety of terms, including child-to-parent-violence (Contreras & Cano, 2016; Holt & Retford, 2013), child-to-parent abuse (Kuay et al., 2017), adolescent-to-parent abuse/violence (Hong et al., 2012; Miles & Condry, 2015), it is generally thought to refer to the use of physical, emotional, verbal, and financial abuse by children to threaten, intimidate and control their parents (Cottrell, 2001; Kennair & Mellor, 2007). CPA is often conceptualized in the literature as an adolescent phenomenon, with perpetrators often reported to be between 14 and 17 years old (Condry & Miles, 2013; Holt & Retford, 2013). However, parent reports indicate that CPA behaviors can be seen as early as five years of age but are often not conceived of as abuse until adolescence when the power imbalance is more prominent.

Lack of recognition of CPA is highlighted by practitioners as being a fundamental problem (Miles & Condry, 2015), resulting in CPA remaining on the periphery of research and social policy agendas. Practitioners hold contrasting conceptualizations of CPA as a justice problem, a child protection issue, or a subtype of domestic violence (Holt, 2016). Within England and Wales, CPA seems to be gaining recognition within the policy arena and is increasingly being conceptualized as a form of domestic violence, which many practitioners are accepting of (Miles & Condry, 2015). In March 2015, the Serious Crime Act introduced a new criminal offence of “coercive or controlling behavior in intimate or family relations,” which may be applied to anyone over the age of criminal responsibility (10 years old) (http://www.legislation.gov.uk/ukpga/2015/9/contents/enacted). This may lead to the prosecution of young people who are aggressive toward their parents. However, although there are similarities between CPA and domestic violence, CPA does not fit neatly within a dichotomous victim/perpetrator framework (Nixon, 2012). A parent–child relationship is very different from an intimate relationship and in most cases, there is no desire to sever the relationship (Miles & Condry, 2015).

The focus of research to date has largely been on developing victim and offender typologies, although this has led to the identification of CPA risk factors, it has also hindered definitions of CPA and how to respond (Moulds et al., 2016). Risk factors of CPA identified by prior research include gender and history of domestic violence. Research into the role of gender has resulted in contradictory findings. Some studies suggest CPA is more common amongst males (Ibabe et al., 2014), others amongst females (Day & Bazemore, 2011), and other studies indicate males and females are equally likely to exhibit CPA (Pagani et al., 2004). Mothers are consistently found to be significantly more prone to be victims of CPA (see Simmons et al., 2018, for a review). A recent meta-analysis highlighted that both being victimized and witnessing domestic violence was a risk factor for CPA (Gallego et al., 2019). In some cases, young people use violence to protect the abused parent (Cottrell & Monk, 2016).

Another factor closely linked to CPA is parenting; with both authoritarian and permissive parenting associated with CPA (Hoyo-Bilbao et al., 2019; Ibabe, 2019) as well as parental substance abuse leading to CPA (Calvete et al., 2020; Svensson et al., 2019). Nowakowski-Sims (2019) found that adolescents who were arrested for violence toward their parents and siblings experienced a high amount of childhood adversity, such as parental substance use, parental imprisonment, and ineffective or absent parenting. Suárez-Relinque et al. (2019) reported that among Spanish adolescents parenting characterized by warmth, nurturance and emotional support was important in protecting against CPA. In line with this, López-Martínez et al. (2019) showed that adolescents with low CPA obtained higher scores for both positive family communication and the ability to control and alter emotions. There are thus contrasting conceptions of CPA as problematic behavior resulting from inadequate parenting or as a form of domestic abuse. A more focused definition would enable a clearer understanding of CPA and identification of its risk factors will inform effective responses.

Research on CPA is often focused on the views of parents, whilst there is a dearth of research looking into the views of practitioners. Practitioner perspectives are important, as practitioners are often the first point of contact and responsible for responding to CPA. A qualitative case study of United Kingdom practitioners from youth justice, domestic violence and child protection fields found their understanding of CPA was often uncertain and conflicting (Nixon, 2012). For instance, social workers were often unfamiliar with the term “parent abuse” and found it challenging to reconcile the idea of abuse perpetrated by young people with their professional notion of “safeguarding children in need.” Instead they preferred to label the behavior as “challenging” or “poor parenting.” In contrast, practitioners from domestic violence and youth justice fields were more accepting of parent abuse as a form of family violence and less likely to view it as a parenting deficit. However, all professionals felt that CPA was associated with earlier experiences of domestic violence. This study though did not outline the methodological approach used, limiting the validity of the findings. Qualitative research is needed within this field to explore conceptions and approaches to CPA, but this research needs to be grounded in a strong methodological approach in order to inform policy and legislation.

A further qualitative study of nine practitioners working with CPA (i.e., police, youth offending, charities) in the United Kingdom found CPA was most frequently perceived as rooted in family dysfunction, including current and past family abuse or poor parenting (Holt & Retford, 2013). Others viewed it as a power imbalance, reflecting the power struggle characteristic of adolescence. Practitioners were less likely to identify proximate psychopathology factors (e.g., mental health issues, substance abuse). Explanations that surround poor parenting skills risk blaming parents and suggesting it is parents who must be the transformative agent. This lack of shared understanding as to who or what is the problem may hinder coordinated working across agencies, which may have implications for developing a more coherent response to CPA.

The lack of a clear and consistent definition of CPA means that professionals often do not know how to respond effectively. Without a clear and consistent understanding of the issue universal policy and legislation cannot be developed on how to effectively manage CPA. This lack of appropriate legislation, policy, and support for families experiencing CPA (Condry & Miles, 2013) has meant a coordinated strategy to address CPA has yet to be developed (Condry & Miles, 2013; Nixon, 2012), which significantly impacts young people and their families. International research into parents’ experiences of CPA has indicated that they feel that the police (Haw, 2010), judiciary service (Eckstein, 2004), youth offending services (Holt, 2009), social services (Hunter et al., 2010), education and health services (Parentline Plus, 2010), and voluntary and community services (Holt, 2011) often fail to respond effectively to CPA. In some instances, involvement of support services can serve to worsen the situation by triggering retribution from the perpetrating child (Pagani et al., 2003) or leaving parents feeling blamed by practitioners (Cottrell, 2001). In the United Kingdom, government guidance for practitioners on understanding and responding to CPA was first published in 2015 (Home Office, 2015). A lack of police policy guiding how to respond to CPA can lead to over reliance on the discretion of the responding officer (Miles & Condry, 2015). Police officers are more likely to proceed with arrest based on the gender of the victim-offender dyad as well as if the victim has sustained injury (Armstrong et al., 2018). Thus, research is greatly needed to facilitate a better understanding of CPA in order to improve system responses and resources for affected families and facilitate effective intervention strategies that do not result in parents feeling blamed.

The limited attention in research and policy directed toward defining and understanding CPA has resulted in inconsistent definitions from different spheres (e.g., criminal justice, domestic abuse, poor parenting) and hindered understanding of risk factors and effective responses, which has a detrimental impact on children and parents. These gaps in the literature are further confounded by the small number of qualitative studies, particularly with practitioners (Gabriel et al., 2018; Miles & Condry, 2015; Nixon, 2012). In-depth qualitative research will enable an exploration of how practitioners, who are working with families experiencing CPA, define and respond to CPA. Building on prior smaller scale qualitative studies, the present study is a qualitative exploration of a range of practitioner’s accounts of CPA. The present study builds on prior work as it involves a larger number of practitioners from a range of fields (e.g., police, charities) and explores not only practitioners’ understandings of CPA but how they address it. The aim of the study is three-fold: to define the problem of CPA, to identify similarities and differences across practitioner understanding of CPA and the risk factors they identify, and to explore how practitioners effectively respond to CPA. The present study will therefore both identify whether prior findings resonate in larger, more diverse samples and explore further avenues. The findings of this project will thus inform both current understanding of CPA, and policy on how to effectively support families experiencing CPA.

## Method

### Participants

A sample of 25 practitioners (19 female and 6 male) working for three nonprofit domestic violence organizations and one police force around the United Kingdom participated in four focus groups, consisting of 5–9 participants each. Participants included youth justice team officers (*n =* 4), project managers, support workers, and volunteers at relevant charities (*n* = 18), an educational psychologist (*n* = 1) a retired police worker (*n* = 1) and a police constable (*n* = 1). Their mean age was 40.44 (*SD =* 13.36) years of age and their average years in the role were 4.7 (*SD* = 3.73). Three participants before going into their current role had worked for other sectors in the police, such as youth justice, child protection, investigation management and two others had worked in the police for 6–12 years. Participants were recruited from the South, South East, and North of the United Kingdom.

CPA/domestic violence support organizations/charities and police stations in England were identified online by the researcher (third author of this study). An email was sent to the organization director or senior member of the police describing the aims of the study and asking if they would be willing to allow colleagues linked to their organization whose work covers the area of CPA to be approached to participate in focus groups on their understanding of CPA. If the director agreed, information sheets were sent to support workers inviting them to take part in a focus group. There was no monetary reimbursement for their time involved and participation was voluntary. The participants provided informed consent and were informed of their right to refuse to participate or withdraw from research.

### Procedure

Focus groups were conducted in-person in the participants’ place of employment, including a police station and in three nongovernmental organization offices. The focus groups were led by one of the authors of this paper and each lasted around 50 minutes. Focus groups were semistructured; participants were asked open-ended questions regarding behaviors, frequency, and risk factors of CPA. The question topic guide was developed based on the findings of prior research within the field. The topic guide was pilot tested before the main data collection phase. The same topic guide was used for all focus groups. Example questions include: what are the key types of behavior CPA involves, what do you think are the risk factors for involvement, what are the key outcomes? Ethical approval was granted by the University Research Ethics Committee.

### Thematic Analysis

This was an explorative qualitative study that used thematic analysis to identify key themes across practitioner interviews. Thematic analysis following the phases of analysis outlined by Braun and Clarke (2006): data familiarization, initial coding, search for themes, review of themes, theme definition, and reporting. The data analysis was completed using NVivo 11. The first author identified codes across transcripts, refined the codes, and generated themes based on these codes. The other authors of the paper then reviewed and revised these themes.

Due to the exploratory nature of this study and the limited prior work in this field, the coding was inductive. The coding framework was developed from the interview data. Both semantic (explicit data themes) and latent (underlying patterns) themes were identified. A semantic approach was adopted in order to capture definitions and understanding of practitioners from their point of view. Latent themes emerged during the analysis in that clear overarching patterns across interviews were apparent.

## Findings

**Figure 1.**
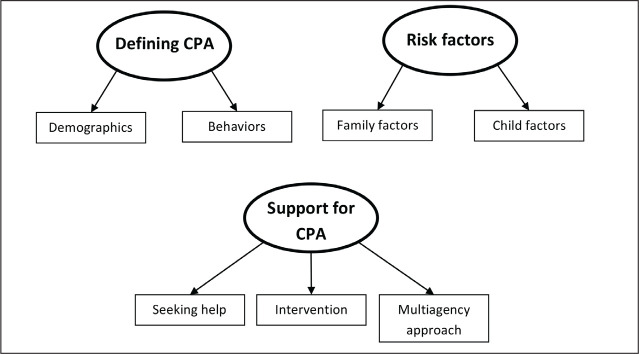
Thematic diagram of practitioner interviews. Main themes are presented in circles and subthemes in squares.

Thematic analysis resulted in the identification of three main themes: defining CPA, risk factors for CPA, and seeking support for CPA (Figure 1).

### Defining CPA

This theme explored how participants understood and defined CPA. There were two subthemes identified: demographics and behaviors.

*Demographics.* Participants reported that they worked with CPA from early childhood to late adulthood. Participants stated that young children’s aggressive behavior is often seen as more acceptable but becomes less so with age as children become stronger:

I’ve worked with families, very young children to young people, young adults, I guess the level of acceptability of the aggression gets less acceptable as they get older, so little children hitting their parents seems to be a bit more okay ... but parents are more accepting of it than perhaps they are when children are older, stronger, able to do more things sometimes. (P5, Educational Psychologist)

Participants further suggested that there were gender differences in CPA frequency and behaviors:

In boys it’s more frequent, girls it’s less, girls it’s more emotional and verbal. Boys is percentage wise, it’s more physical and emotional and financial. (P17, Project Manager)

Participants generally felt that boys engaged in more physical aggression and girls in more emotional aggression. One participant also reported that girls are more likely to use weapons.

*Behaviors.* Participants felt that CPA included not just physical acts of aggression, but damaging property, verbal aggression, threatening behavior, emotional control, and financial abuse:

I’d say there’s quite a big range for me that comes under that sort of umbrella, that could be more physical acts of aggression but also could include, for example, the verbal aggression but also there’s sort of threats of aggression perhaps, that are against property or money or all of those kinds of things that come, it’s almost a bit more controlling or attempting to control sometimes, parents’ behaviour. (P5, Educational Psychologist)

The majority of participants felt that property damage, verbal aggression, and emotional control were the most common types of behavior and that physical aggression and use of weapons was rarer.

Participants often discussed a progression from more verbal aggression to physical aggression. This progression was often reported to occur as children developed with age:

We work from aged 6 to 18, so we do get a lot of referrals from primary schools. They’re more preventative measures, they haven’t quite reached the stage where they become physical. Some of the little ones are when they’re kicking, spitting, hitting, but it’s usually verbal isn’t it? (P17, Project Manager)

CPA in adulthood was often reported by participants to involve financial abuse.

Participants felt that for aggressive behavior to be defined as CPA and be reported it had to be a frequent occurrence that extended beyond normal rule breaking:

It’s normally an ongoing thing, it has to be for us to take the referral, so we’re not looking at just kids being teenagers and breaking rules and boundaries and so on, it has to be something more than that... (P16, Project Manager)

### Risk Factors

This theme outlined the underlying reasons participants attributed to the development of CPA. There were two subthemes identified: family factors and child factors.

*Family factors.* Participants identified a range of underlying reasons for CPA that were related to the family, including family structure, parent mental health, and parenting.

History of domestic abuse was a central risk factor for CPA identified by participants. Young people who engaged in CPA were reported to have often experienced a range of abusive situations, such as witnessing domestic abuse or being the targets of abuse themselves. Participants felt that children learned and imitated CPA behaviors. Participants also stated that parents who had been victims of domestic abuse may find being assertive with their children challenging:

…we’ll have parents who’ve had an abusive background, where they didn’t have anything so they over compensate by buying the children, “They’re not going to feel like I did” or where they’ve been abused or they’ve hit their children, but they don’t know how to be assertive with the children and do positive parenting and so they let the children get away with more… (P10, Service Manager)

Another reason suggested by participants was mental health issues within the family, be this the parent or the child themselves. Participants felt that parents who were suffering from mental health issues may find being able to effectively parent and manage their children’s needs challenging:

People who have depression, bipolar, substance abuse, those kinds of issues have been in the family and therefore have made it more challenging for them to be able to consistently support their child’s emotional needs so sometimes it becomes difficult for that child to be able to say, “This is what I’m angry about” and it becomes more physical and more verbal in that way. (P8, Domestic Abuse Advocate)

A few participants discussed that permissive parenting and a lack of boundaries can lead to CPA. In particular, participants indicated that children, even young children, are exposed to conversations that are not appropriate in a parent–child relationship, which may cause strain on the relationship:

I think if a child is exposed to sort of too much from their parents, they will start to respect their parent less, if their parent is talking to them about problems or issues that they’ve got or issues around the house, a child will stop seeing their parent as being the one that’s caring for them, the one that’s responsible for them, the one that will solve their problems and that can potentially lead to a lack of respect and sort of boundaries being displaced... (P8, Domestic Abuse Advocate)

Although participants did not specifically blame parents for CPA, there was a general theme that CPA surrounded parenting skills.

*Child factors.* Some participants felt that special educational needs (SEN) was a central factor in CPA:

I think probably the majority of the families we come across where there’s aggression towards the parent, it’s because of additional needs of some description and not necessarily intentional but because of lack of understanding. (P7, Senior Coordinator)

However, other participants viewed SEN labels more negatively and felt that behavior, not diagnosis, was more important.

Participants felt that some children who exhibited CPA did not fully understand emotions or have full control over their emotions. Participants felt that children were frustrated as they were not able to express themselves and this resulted in aggression:

Maybe for the child, they’re frustrated because they can’t express whatever their emotion is, they don’t understand it so then the parents are frustrated and it just goes from there. (P2, Young Persons Manager)

This questions whether CPA refers to intentional behavior or also includes CPA due to SEN or poor emotional control.

A number of participants felt that CPA and criminal behavior were intertwined. For example, drug use was thought to be related to CPA by a few participants, especially where the child required money to purchase drugs. In addition, gang involvement was mentioned by a sample of participants, mainly from youth justice and the police. In particular, the financial element of gang behavior was highlighted; be that the gang providing material resources or the young person owing the gang money. Children may become aggressive toward their parents when they owe the gang:

…you think as well with the gangs, say like the mum or dad takes something away from them that they may owe to the gang, then that young person has the fear of what’s going to happen to them and the repercussions it’s going to have on them. That’s probably where some anger stems from. (P20, Youth Justice Officer)

There was thus some lack of consistency across practitioners from different fields regarding the development of CPA.

### Support for CPA

This theme explored support offered to families experiencing CPA. There were three subthemes: seeking help, intervention, and a multiagency approach.

*Seeking help for CPA.* This subtheme surrounds reporting CPA, including services parents can report to, lack of awareness of how to manage reports of CPA, and stigma associated with reporting CPA. Participants had mixed feelings regarding reporting CPA to the police. For example, some participants stated that they encouraged parents to report their child to the police, while other participants indicated that the police were not an effective solution and parents may be reluctant to do so:

…I mean people don’t want their children to have a criminal record because then obviously when they try to get a job, there’s all these repercussions. (P1, Chair Domestic Abuse Forum)

Participants indicated that there are stereotypes associated with social services and they are often viewed negatively by families:

And there’s huge stereotypes to come to terms with, we did some training recently with Children’s Services and being blunt, they’re seen as the enemy, when someone gets a phone call from Children’s Services, they stop listening after those words come out on the phone, it’s just white noise after that point and they think someone is going to come in and take their children away. (P4, Director)

Participants indicated that social services may encourage parents to report their child to the police, especially where there are siblings in the house.

There was a latent theme that social services were not supportive of parents.

Participants suggested that parents are more likely to report older children and boys for CPA as they more often use physical aggression and are more intimidating. Added to this, the presence of siblings was suggested by participants to increase parents’ willingness to report CPA:

I think severity of damage and intent and I think contributing factors would be other siblings being in the house, so obviously them being at risk and I think again from personal experience, it tends to be, if it’s only being directed at the parent and there’s been no-one else involved, the parents more, well less likely to report it but if there’s been another sibling there or if there’s been potential harm to another sibling, then obviously they will report that to protect the other child, in my experience. (P8, Domestic Abuse Advocate)

Some participants also stated that is not always parents that report CPA, but other organizations like schools may pick up on signs:

To some extent, it’s also taken out of a parent’s hands because another place where it manifests itself is in schools and if a school is reasonably progressive and modern and is supported by the LEA and all those good things, they’ll have people that start to notice the behaviour trends and it may not be linked to anything to do with DV or DA but some sort of warning bells and generally, a meeting with the parents may start to break those barriers down, so it may not totally come from the parents, it might be another agencies and another one is through the doctor, most people take the soft option of talking to a doctor first rather than anyone more official like police or support workers. (P4, Director)

Participants felt that parents were not often aware they were victims of CPA, especially when perpetrators were girls, and may consequently not seek support. Participants suggested that parents needed to be better educated about CPA and how to recognize it.

There were mixed thoughts, even amongst participants from the same or similar fields, regarding the role of ethnicity and culture. Some participants felt that language barriers or a less accepting attitude toward SEN may make reporting CPA challenging. Other participants felt that CPA occurred across ethnicities and cultures:

I think if you’d have asked me this question a few years ago I would have said yes. If you’re asking me the question about currently I’d say (culture has) got nothing to do with it. (P17, Project Manager)

In some ways, in some cultures, children are treated as children and not allowed to disrespect the elders so potentially, it might be that there’s less? (P1 Chair Domestic Abuse Forum)

A central theme associated with reporting CPA was stigma. Participants suggested that parents may be reluctant to seek help for CPA as they fear the stigma of being considered a “bad parent.” Participants said that parents may feel ashamed and blame themselves and that these feelings may build. Consequently, parents become concerned that reporting CPA will result in their children being removed from their custody:

…at the heart of it there’s a parent/child relationship which parents feel lots and lots of things in response to what’s happening, some of them being guilt and shame about how it’s got to this stage, sort of blaming themselves or for something they might have done, worried about the potential consequences of actually saying it out loud, what will that mean, will it mean that someone is going to come and take them away and all of those really difficult feelings that makes it incredibly hard to even say there’s an issue… (P5, Educational Psychologist)

There was thus a latent theme of parents not wanting to be seen as “bad parents.”

*Intervention.* Participants discussed the lack of availability of services to support families experiencing CPA, especially services for children under 16 years old. Some participants stated that improved access to mental health services for young people is greatly needed, whereas other participants suggested that more services specifically targeting CPA are needed. Where CPA services were available, they were often limited by resources, meaning a number of families were not able to access support. Participants felt that rather than a reactive approach to CPA they should be more proactive and focus on early intervention.

Participants felt that an effective approach to intervening in CPA cases was holistic (dealing with all aspects of the situation), direct, and solutions focused:

…when we first started, because we weren’t using solution focused therapy. It was about, “Oh what happened, why are you hitting your mum? … and it just wasn’t working, because we’re dictating what they should be doing. We never ask, “What do you want instead, what’s happening?” Because if you don’t know your destination, how do you know how to get there? That’s how we are driven at the moment, it’s, “Right yeah he’s hitting you, but what do you want him to do instead?” “I want a hug,” and the child is sitting there listening. “Well what difference would a hug make?” “I’d know that he loves me back,” and the child is sitting there. They’re far more likely to be thinking about hugging their mum than with me saying, “Why don’t you hug her?” (P17, Project Manager)

Participants felt that a key aspect of managing CPA effectively was developing consistent boundaries but acknowledged it may be challenging for parents to set boundaries with their aggressive children.

A key theme across participant interviews was the need for supportive relationships. Participants said that they support parents and build their confidence:

…It was just picking [the mother] up, it’s okay to have a bad day. What are we going to do to make it better? Just acknowledge it. And when the parent feels confident, as confident as they can be, they cope better. (P17, Project Manager)

However, some participants discussed difficulties surrounding engaging children and their families in CPA support:

She and the son will not engage, not just with us but with no one. You phone the college and he’s say, “Yeah I’ll meet you there tomorrow at 12,” he doesn’t turn up. You go to the house, he doesn’t open the door. (P16, Project Manager)

Again, there is an underlying theme here that ineffective parenting is a root cause of CPA and parents should thus be the transformative agents.

*Multiagency approach.* Participants said that they communicate with other organizations and provide advice on supporting victims of CPA, but felt a multiagency approach to supporting families was not possible:

I mean we do meet with a lot of outside agencies in our team around the family meetings, social care meetings, so we do have a good network, and we do have a lot of people calling us all over the UK for advice … so, in that respect there is a lot of dialogue. In terms of working together, that’s near enough impossible, because we use solution focused therapy here and you won’t find services really ... well in the UK where child to parent abuse where they use solution focus. (P17, Project Manager)

Participants expressed a desire to work with other agencies, such as social services, to provide support to families experiencing CPA. However, they felt that lack of resources or consistent approach hindered this.

## Discussion

This qualitative study explored the perspectives of practitioners from diverse backgrounds, including the police, youth offending, counselling, and charity backgrounds on CPA. Thematic analysis of practitioner’s interviews identified three central themes, relating to definitions of CPA, understanding of CPA risk factors, and seeking help for CPA.

### Defining Child-to-Parent Aggression

Practitioners from a range of backgrounds had similar conceptions of CPA that were much broader than previous research definitions. Based on the findings of this research, the following definition of CPA was developed:

Aggressive (physical, verbal, emotional), threatening, or abusive (financial) behavior used by a child of any age repeatedly to intimidate or control a parent.

In line with prior definitions (Cottrell, 2001), practitioners referred to the use of physical, emotional, and financial abuse. However, practitioners also referred to verbal aggression, threats using weapons, and property damage. Practitioners defined CPA as the use of aggressive or threatening behavior to intimidate or control parents and that parents felt was beyond their control. Although practitioners’ conceptualizations of CPA were broader than research definitions, it was highlighted that CPA was a recurrent behavior that went beyond “typical” rule breaking behavior, in line with findings from Simmons et al. (2019) in Australia. Specific behaviors may need to be identified for research purposes, but in policy and practice definitions of CPA may be broader and more focused on the purpose and impact of the aggressive behavior on the parent. This broader definition of CPA of involving a range of aggressive behaviors fits with the Serious Crime Act (2015) offence of coercive or controlling behavior in a family relationship recently introduced; reflecting the trend toward a domestic violence perspective of CPA.

In contrast to conceptions of CPA as being an adolescent phenomenon (Hong et al., 2012), practitioners viewed CPA as occurring across the lifespan. Previous research has typically used police data to assess the age at which CPA occurs (Condry & Miles, 2013), which may result in a skewed perspective as this data will only refer to reported cases. Parents may not report CPA until adolescence when children are physically stronger and CPA behavior may become more severe and be seen as abusive (Ulman & Straus, 2003). Indeed, practitioners reported that CPA behaviors may develop from manipulation in early childhood to financial abuse in adulthood. Greater research attention needs to be directed toward understanding the progression of CPA across the lifespan. The present study therefore supports the argument of Simmons and colleagues (2018) that CPA definitions need to move away from a focus on child age and instead focus on the parent–child relationship.

### Understanding of Risk Factors for Child-to-Parent Aggression

In contradiction to research that has found that practitioners’ understanding of CPA is often uncertain and conflicting (Nixon, 2012), practitioners appeared to have largely consistent understandings of CPA risk factors. This may be the result of emerging literature and guidance on CPA (Home Office, 2015). In line with prior work (Contreras & Cano, 2016; Gallego et al., 2019; Ibabe et al., 2014), domestic violence was suggested to be a main risk factor for CPA. Again, this may reflect the growing consensus that CPA should be understood within a domestic violence framework (Miles & Condry, 2015). Another central risk factor highlighted by practitioners was parenting (Suárez-Relinque et al., 2019). Even practitioners from a youth justice/police background indicated that parenting was a factor and felt sometimes the police were used as a parenting tool, contradicting prior research that has suggested those from a youth justice background are more reluctant to conceive of CPA as the result of parenting (Nixon, 2012). Building on this prior work, practitioners felt that permissive parenting resulted in a lack of boundaries and exposing children (even during early childhood) to conversations that were not appropriate for the parent–child relationship. Exposure to inappropriate conversations was suggested to blur the boundaries and respect between the child and parent.

In contrast to a previous qualitative study (Holt & Retford, 2013), practitioners often identified psychopathology risk factors. Practitioners indicated that parents of children demonstrating CPA often experienced mental health issues, and this reduced their ability to manage and support their child’s behavior. Building on this, children’s emotional development was also thought to be an important risk factor. According to practitioners, children demonstrating CPA find understanding and managing their emotions challenging. Poor social information processing has been found to predict greater CPA (Orue et al., 2019). Children who become frustrated may be more likely to attribute hostile intent to their parent’s actions and consequently may behave aggressively. The role of children’s emotional and social processing in CPA requires further investigation.

Related to this is the issue of intent. In the literature CPA has been defined as behavior that is intended to cause harm to parents (Calvete et al., 2013). However, there was inconsistency amongst practitioners in the present study as to whether aggression toward parents had to be intentional or not to be considered CPA. Some practitioners felt that children with SEN engage in CPA, but that their behavior is not intentional but the result of their condition. Added to this, poor emotional understanding and control may result in CPA, but it was questioned whether the child’s intent was to harm the parent or a reaction to frustration. This may influence whether the behavior is classed as CPA and the way in which practitioners respond.

In line with a small-scale study of practitioners within police, youth offending, and charity fields (Holt & Retford, 2013), some practitioners identified patterns across CPA cases and others did not. For example, some practitioners felt that there was no gender difference in CPA involvement, whereas others felt boys were more likely to use physical aggression and girls emotional aggression. Added to this, there were contrasting views on whether there were differences across cultures and ethnicities. These contrasting views may represent practitioners’ varied backgrounds and experiences of CPA. There has been limited work on the cultural variations in CPA. Emerging evidence indicates that it is a global problem (Coogan, 2012; Miles & Condry, 2015), but whether the same understanding and risk factors are identified across cultures is poorly understood. The present study therefore indicates that CPA needs to be understood from multiple perspectives, including psychopathology and family factors.

### Responding to Child-to-Parent Aggression

Practitioners in the present study were working within the field of CPA and were therefore aware of CPA. However, practitioners reported that parents sometimes do not realize they are experiencing CPA and do not know who to seek support from. The limited reference to CPA in policy and the limited support available likely leads to this lack of recognition. This problem is further confounded by the fact practitioners felt parents were often reluctant to seek help for CPA either because they felt stigma or blamed for their child’s behavior (Cottrell, 2001) or because they were concerned of further CPA (Pagani et al., 2003). Focusing on understanding and intervening in CPA in research and policy may consequently raise awareness of this issue and reduce the stigma associated with seeking help. Added to this, research needs to identify effective ways of responding to CPA that do not put the parent at risk of further harm. Practitioners also identified that in some cases CPA cases are picked up by those external to the family, such as schools. Educating individuals who come into contact with children and families, such as school staff, health practitioners, children’s club workers, to identify the risks and signs of CPA may enable more families to be able to gain support. This reduces the onus on parents to report CPA, especially where they are unaware of CPA or are reluctant to seek support.

The present study supports the finding that there is a lack of a coordinated strategy to address CPA (Hunter et al., 2010; Miles & Condry, 2015) and that practitioners are unsure of the most effective way to respond to CPA (e.g., Haw, 2010; Hunter et al., 2010). Practitioners were unsure which service parents should report CPA too and many felt these services were not well equipped to deal with CPA. For example, many practitioners felt that reporting the child to the police was not an effective strategy as many parents were reluctant to do so for fear of the consequences for their child (e.g., criminal record). Further, police interviewed in the present study felt that they were sometimes used as a parenting service and there was little support they could offer for CPA. In the absence of police policy on responding to CPA, many police officers may be required to rely on their own discretion on how to respond (Miles & Condry, 2015) and as a result may be influenced by factors such as child gender and neighborhood characteristics (Armstrong et al., 2018), which may leave some families without the support they require.

The other main option practitioners discussed for parents reporting CPA was social services. Although some practitioners felt that social services should be a viable option for parents, practitioners felt that their resources were limited to support families and that this route may result in child protection issues. Practitioners felt that there was a stigma attached to social services, which they appeared to also hold themselves. Further, parents often do not wish to sever the parent–child relationship (Miles & Condry, 2015) and consequently be reluctant to engage with social services. Some practitioners suggested the need for more mental health services and specific CPA support organizations to support families. The current conceptualization of CPA as a subtype of domestic violence may be hindering this approach and promoting a police / social services approach. Parents may be more willing to seek support from services where they feel they are less likely to be judged and where there will not be repercussions for their child. The current findings thus support the view that a multiagency, coordinated strategy to effectively respond to CPA is needed (Condry & Miles, 2013). However, the present study revealed that some practitioners do not feel this is possible as their services do not fit with other agencies. Policy should thus focus on how agencies can work together in a coordinated way.

Previous research into CPA has yet to explore effective methods of intervening. Practitioners felt that the most effective way to intervene in CPA was solutions focused therapy. Solutions focused therapy is a brief therapy approach that focuses on solution building rather than problem solving (Macdonald, 2011). The focus of the therapy is on using individuals own resources rather than on problems and past causes. Further, practitioners highlighted that interventions are only effective when all aspects of a situation are taken into account. They felt you needed to work with both the child and parent and build a supportive working relationship with families. Identifying how to effectively intervene in CPA has not received sufficient research attention. This is greatly needed when developing a strategy to respond to CPA as once parents have reported CPA this needs to be dealt with effectively so as not to worsen the situation (Pagani et al., 2003), make parents feel blamed (Cottrell, 2001), and to prevent the CPA and the implications this has for families (Kennair & Mellor, 2007).

### Strengths and Limitations

This was a qualitative study of practitioners from a diverse range of fields. This study builds on limited prior work relating to how those at the frontline of CPA understand and respond to CPA. The qualitative design of this study enabled a more in-depth picture of practitioner understanding to be developed and provided novel insights, such as how to effectively intervene in CPA cases. The sample was diverse in terms of background, experience and gender of practitioners. Further, focus groups were held across the United Kingdom meaning a regionally diverse sample was recruited. However, practitioners in the present study were currently working with CPA cases and consequently findings may not apply to practitioners who have limited or no experience with CPA. Possible limitation could be that: the findings of the current study are culturally and geographically specific. However, recent evidence indicates that it is a global problem (Miles & Condry, 2015; Simmons et al., 2019; Suárez-Relinque et al., 2019). The understanding of CPA and its risk factors may be further investigated in different cultural contexts. The diverse range of participants also limited the ability to make comparisons across views from different sectors. Further research may also consider more in-depth analysis such as conversational analysis.

## Conclusions and Implications

Practitioner definitions of CPA were broader than research definitions, encompassing a wider range of aggressive behavior. Practitioner definitions were less focused on child age and more concerned with the intimidating and controlling nature of the behavior. Policy definitions of CPA, in contrast to research definitions, may consequently need to reflect the range of behaviors and their uses. However, the role of intent needs to be further explored in future research. Practitioners felt risk factors for CPA reflected psychopathology, domestic abuse, parenting, and family dynamics. However, those with a youth justice / police background also highlighted the role of gangs. This indicates that approaches to understanding and intervening in CPA need to understand CPA from a multilevel perspective. Practitioners also highlighted issues surrounding reporting CPA and felt that the current avenues of the police and social services were ineffective. This research highlighted the need for a multiagency coordinated strategy to address CPA, but practitioners did not always feel this was possible. Policy thus needs to focus on identifying how agencies can work together in the most effective way. Further, the current study found that a solutions and family focused approach to intervening in CPA cases may be effective. Greater research attention is needed to identify the most effective way to intervene in CPA and improve outcomes for vulnerable parents and children. The present study emphasizes the complexity of defining and understanding CPA, especially as it crosses multiple sectors (e.g., police, social services, charity).Working toward a consistent, multiperspective understanding of CPA will enable a more effective approach to CPA and consequently more effective support for victims.
